# Strain-Rate-Dependent Tensile Behavior of Carbon and Aramid Fiber Bundles: Experiments and Constitutive Modelling

**DOI:** 10.3390/ma19214610

**Published:** 2026-10-30

**Authors:** Ziyi Xue, Jia Qu

**Affiliations:** Key Laboratory of Advanced Composite Materials Structure and Mechanics, College of Aerospace Engineering, Harbin Engineering University, Harbin 150001, China; xueziyi@hrbeu.edu.cn

**Keywords:** carbon fiber bundle, aramid fiber bundle, strain rate effect, Weibull distribution, Ramberg–Osgood model

## Abstract

**Highlights:**

- TZ300 carbon fiber bundles exhibit negligible strain-rate sensitivity in tensile strength over 10^−4^ s^−1^ to 10^2^ s^−1^.
- P240 aramid fiber bundles exhibit a 60.5% strength increase and transition to brittle fracture at high rates.
- The Ramberg–Osgood model fits all curves with a unified carbon parameter set and a rate-dependent aramid *K*.
- These results provide a rate-dependent modeling basis for the dynamic design of fiber-reinforced composites.

**Abstract:**

Fiber-reinforced composites are widely used in lightweight structures, making the strain-rate-dependent behavior of reinforcing fibers essential for reliable design. This study investigates the tensile response of TZ300 carbon and P240 aramid fiber bundles over a strain-rate range of 10^−4^–10^2^ s^−1^ using quasi-static tensile, pneumatic impact, and split Hopkinson tensile bar tests. The results are analyzed with a Weibull statistical damage model and an explicit saturation-form *R–O*-type model. Carbon fiber bundles exhibit negligible strain-rate sensitivity: tensile strength remains between 3375 and 3496 MPa, while the failure mode changes from progressive to nearly simultaneous fracture. Aramid fiber bundles show pronounced positive strain-rate strengthening: tensile strength increases from 1950 to 3129 MPa (about 60.5%), failure strain decreases from 0.046 to 0.020, and the failure mode transitions from quasi-ductile splitting to brittle fracture. Weibull parameters remain nearly constant for carbon fiber bundles but vary markedly with strain rate for aramid fiber bundles. The Ramberg–Osgood model fits all stress–strain curves well; a unified parameter set describes carbon fiber bundles, whereas the aramid parameter *K* increases exponentially with the logarithm of the dimensionless strain-rate ratio $\dot{\varepsilon }/{\dot{\varepsilon }}_{0}$. At the strength-prediction level, this description is more accurate for aramid than the Johnson–Cook law and physically more informative than the Cowper–Symonds law. These findings indicate that, within this strain-rate range, the fiber strength rate effect can be neglected for carbon fiber bundles, whereas aramid fiber bundles require explicit rate-dependent constitutive models in dynamic design. These results provide a reference for the dynamic design of their composites, where matrix and interface effects should be considered separately.

## 1. Introduction

Fiber-reinforced composites are widely used in aerospace, ship structures, and protective equipment because of their high specific strength, excellent fatigue resistance, and design flexibility [1,2,3]. In service, these structures frequently experience dynamic loads over a wide range of loading rates and strain rates, such as impact, crash, and ballistic events [4,5,6]. As the primary load-bearing phase, the strain-rate-dependent mechanical behavior of the reinforcing fibers directly governs the overall response and failure of the composite; systematic tensile characterization of reinforcing fibers across a wide strain-rate range is therefore essential for the dynamic design and performance prediction of composite structures.

Previous studies have provided valuable insights into the strain-rate sensitivity of fibers. Carbon fibers are generally considered rate-insensitive, with tensile strength changing only slightly with strain rate [7,8]. In contrast, aramid fibers exhibit pronounced positive strain-rate strengthening, with significant strength increases and failure-mode changes as the loading rate increases [9,10]. Moreover, a fiber bundle contains thousands of filaments, and its failure process is statistically controlled by the strength dispersion and defect distribution of the individual filaments [11,12]. Most existing studies, however, focus on either quasi-static or high strain rates, while the intermediate strain-rate range (1^0^–10^1^ s^−1^) remains comparatively less explored. In addition, specimen geometries, clamping methods, and data-reduction procedures differ among studies, making direct quantitative comparison across fiber types difficult [13,14]. A systematic comparative dataset obtained under a consistent experimental protocol is therefore valuable: carbon and aramid fibers differ fundamentally in their failure mechanisms, and comparative testing under identical conditions can further enrich the understanding of their rate-dependent behavior [9,10], as well as supporting engineering material selection and constitutive modeling.

For theoretical description, the Weibull statistical distribution model has been widely used to characterize fiber strength dispersion and damage evolution [15,16]. The Ramberg–Osgood (*R–O*) macroscopic empirical model describes the nonlinear stress–strain response of materials in a concise form [17,18] and has been applied to fiber bundles and fiber-assembled materials. Extending the verification and parameterization of these models across a wide strain-rate range, particularly by combining a statistical damage description with a rate-dependent constitutive model, remains a worthwhile task.

To address these aspects, this study selects representative TZ300 carbon and P240 aramid fiber bundles and uses quasi-static tensile testing, a pneumatic impact device, and a split Hopkinson tensile bar (SHTB) to cover a strain-rate range of 10^−4^–10^2^ s^−1^. Stress–strain curves of the two fiber bundles at different strain rates are obtained systematically. The Weibull statistical model and the explicit saturation-form *R–O*-type model are used for parameter identification and analysis. The aim is to reveal the differences in strain-rate sensitivity, damage evolution, and constitutive description between the two typical high-performance fibers, and to provide reliable experimental data and theoretical support for the dynamic design of fiber bundles as a reference for the cross-scale analysis of fiber-reinforced composites.

## 2. Materials and Methods

### 2.1. Materials and Specimen Fabrication

The TZ300 carbon fiber bundle (abbreviated as CF) was supplied by Weihai Guangwei Composite Materials Co., Ltd. (Weihai, China), and the P240 aramid fiber bundle (abbreviated as AF) was supplied by Zhongfang New Materials Technology Co., Ltd. (Dongying, China). Both have a 3 K specification. The basic material data are summarized in Table 1. No surface treatment or coating was applied before testing, so the fibers remained in their original condition.

The quasi-static tensile specimens were prepared from the original carbon and aramid fiber bundles, which were directly clamped at both ends using a fiber-specific tensile fixture. For the dynamic tensile specimens, both ends were connected to metal threaded connectors to ensure reliable fixation with the testing equipment. Each metal connector had a length of 20 mm and an outer diameter of 2.4 mm. Axially, each connector was machined with an approximately 2 mm non-through hole and a 0.6 mm fiber hole. To ensure effective load transfer at medium to high strain rates and to prevent slippage at the clamped ends, the dynamic specimens were reinforced with epoxy resin, while the intermediate gauge section remained uncured. The epoxy was used solely to anchor the fiber ends inside the connectors: it was injected only into the ~2 mm holes and, after curing, formed an end cylinder of approximately 2 mm in diameter; the gauge section was completely free of resin. The detailed preparation procedure was as follows: an appropriate length of fiber bundle was selected and its surface was cleaned. The two ends of the treated fiber bundle were then inserted through the 0.6 mm fiber holes into the approximately 2 mm non-through holes of the specially designed metal connectors. Epoxy resin (MH10-M5, Senyu Instrument Equipment Co., Ltd., Shenzhen, China) was injected into the approximately 2 mm non-through holes to ensure that the resin fully impregnated and encapsulated the end fibers, so that a cured end cylinder of approximately 2 mm in diameter was formed after curing.

**Figure 1:**
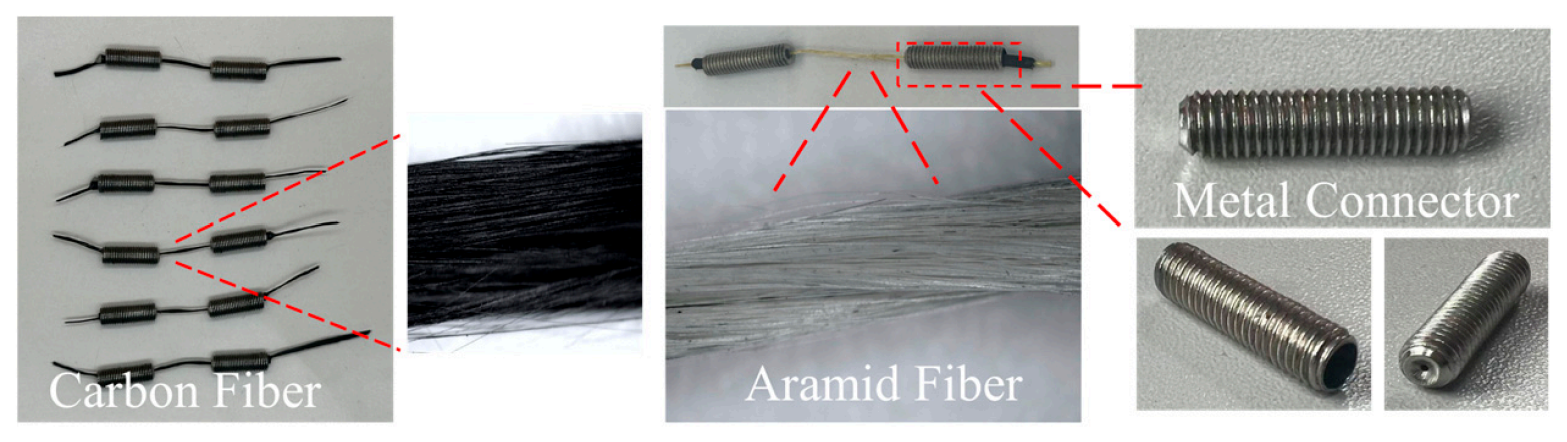
Photograph of the prepared fiber bundle specimens.

The epoxy is a slow-curing room-temperature system with a long working time, which facilitates uniform fiber arrangement. During preparation, the fiber bundle was carefully combed to maintain a uniform distribution of individual filaments and reduce stress concentration, and the specimens were then cured at room temperature for more than 48 h until they fully hardened. The reliability of the present end preparation process was further examined by high-speed camera images, which showed that the cured ends and metal connectors remained intact during loading. Moreover, in all valid dynamic tests, the specimens fractured within the gauge section, and no failure initiated at the epoxy end-tabs or the metal connectors, confirming that the end reinforcement affected only the load transfer and not the measured gauge-section response. During aramid specimen preparation, the fibers were dried at 40 °C for 24 h until reaching a constant weight, and then stored in a desiccator. Each specimen was taken out just before testing, and all tests were completed within 2 h. The testing environment was approximately 25 °C and 75% RH. The metal connectors and the prepared specimens are shown in Figure 1.

### 2.2. Experimental Characterization

**Figure 2:**
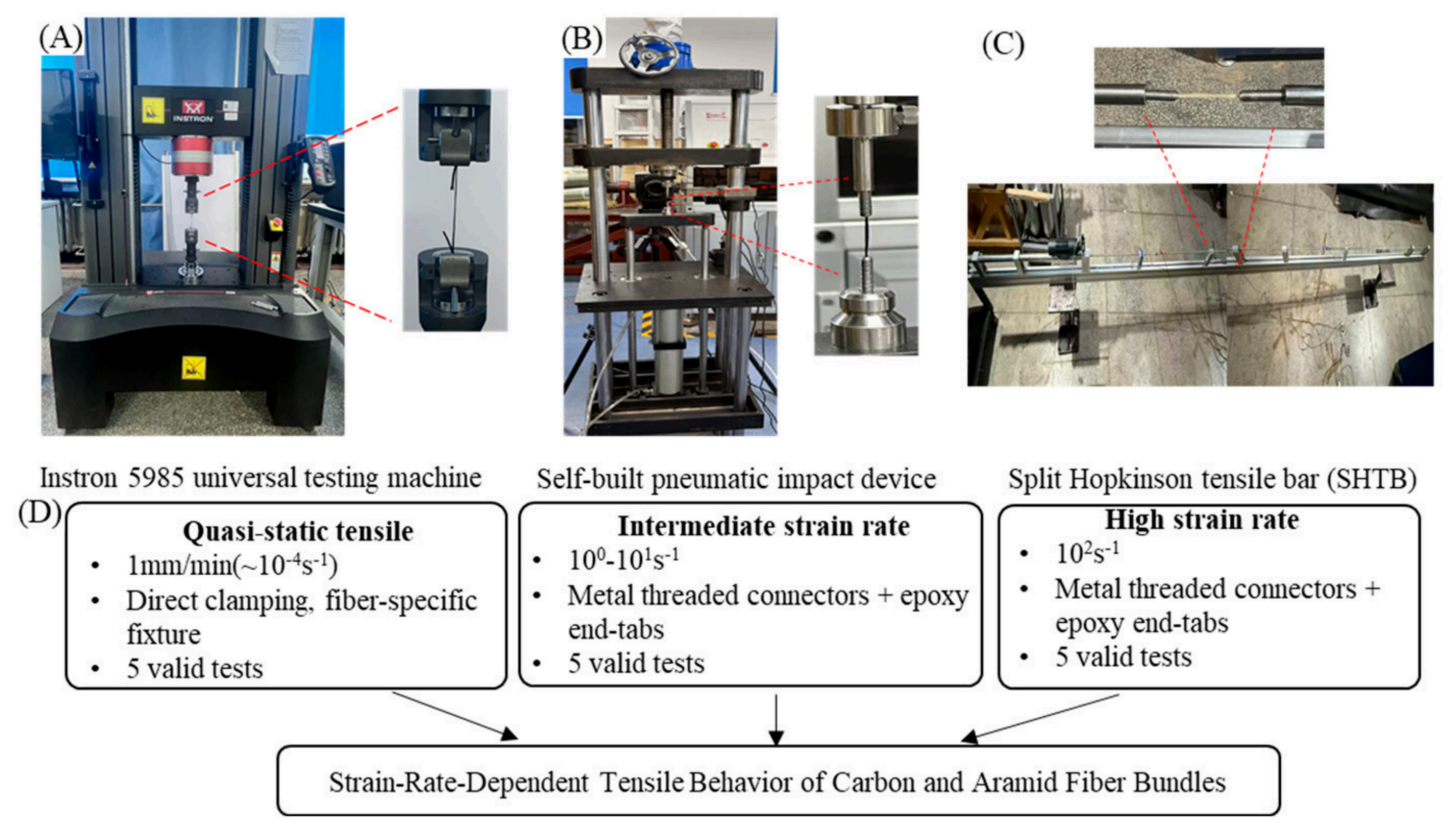
(**A**–**C**) Schematic diagrams and photographs of the testing systems: (**A**) Instron 5985 quasi-static tensile test; (**B**) self-built uniaxial pneumatic impact tensile device; (**C**) split Hopkinson tensile bar; (**D**) flowchart of the experimental program (test setups, specimen configuration, and number of valid specimens per strain-rate group).

Quasi-static tensile tests of the fiber bundles were conducted at room temperature using an Instron 5985 universal testing machine equipped with a ±10 kN load cell (resolution: 0.02% of full scale, ~2 N; accuracy: ±0.5%) and a crosshead position accuracy of ±0.02 mm. A dedicated fiber tensile fixture with textured clamping surfaces was used to prevent slippage and premature fracture at the clamped ends, and its coaxiality was calibrated before testing to ensure pure axial tension. The crosshead displacement rate was 1 mm/min, corresponding to a nominal strain rate of 10^−4^ s^−1^, and data were acquired at 1 kHz. The setup is shown in Figure 2A.

Dynamic tensile properties of the fiber bundles at intermediate strain rates were tested using a self-built uniaxial pneumatic impact device (Figure 2B). Both ends of the specimen were fixed to the static jaw and the dynamic impact head through metal threaded connectors. After the pneumatic valve was triggered, high-pressure gas drove the impact head downward, subjecting the specimen to dynamic tension. The impact speed was controlled by adjusting the intake pressure to achieve the target strain rate. The tensile force was measured by a DYMH-103 force sensor (capacity 100 kg, ≈981 N; output sensitivity 1.0–1.5 ± 10% mV/V; response frequency 10 kHz) installed at the static jaw end and coaxial with the specimen. The axial displacement was measured in a non-contact fashion using a Panasonic HG-C1000 series laser displacement sensor (model HG-C1100, center distance 100 mm, range ±35 mm; repeatability 70 μm; nonlinearity ±0.1%; response time 1.5 ms selectable), mounted on an independent rigid support, with its laser beam perpendicular to reflective markers on the specimen gauge section. The force and displacement signals were recorded using a CONV INV3065N2 data acquisition system at a sampling frequency of 50 kHz.

High-strain-rate tensile tests were performed using a split Hopkinson tensile bar (SHTB) system at strain rates in the order of 10^2^ s^−1^ (Figure 2C). The system consists of an incident bar (3000 mm long, 8 mm diameter), a transmission bar (2800 mm, 8 mm), and a striker bar (20 mm), all made of high-strength steel. A tensile stress pulse was generated by the striker impacting the flange of the incident bar. The incident bar was instrumented with a 120 Ω resistance strain gauge (gauge factor 2.0), and the transmission bar was instrumented with a 120 Ω semiconductor strain gauge (gauge factor 50). The strain signals were conditioned with a bridge excitation of 5 V and an amplifier gain of 100, and recorded using a Donghua data acquisition system at an effective sampling rate of 12.5 MS/s with a 20 k-point record. The nominal strain gauge response time is in the order of 1 μs. For each strain-rate group, five valid tests were performed for both fiber types (see the flowchart in Figure 2D); specimens showing slippage, clamping fracture, or abnormal signals were discarded and replaced.

**Figure 3:**
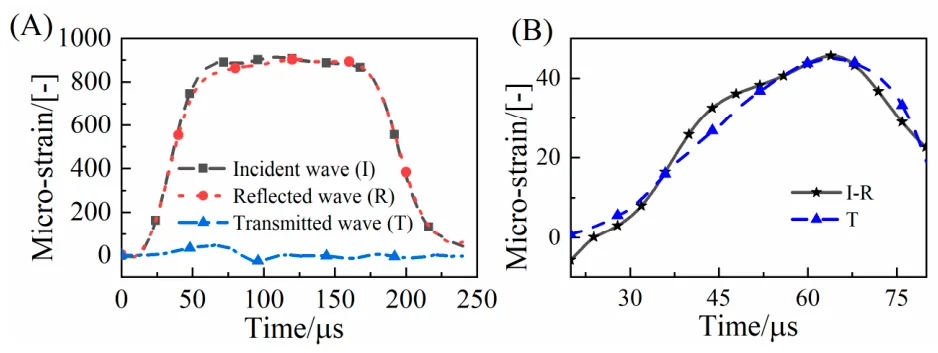
Dynamic stress equilibrium analysis: (**A**) incident, reflected, and transmitted strain–time histories; (**B**) comparison between the transmitted wave and the superposition of the incident and reflected waves.

Representative SHTB waveforms are shown in Figure 3: Figure 3A shows the strain–time histories of the incident, reflected, and transmitted pulses, and Figure 3B compares the sum of the incident and reflected waves with the transmitted wave. The superposition of the incident and reflected waves agrees well with the transmitted wave over the main loading pulse (after a short initial rise time), indicating that the specimen attains dynamic force equilibrium during the essential part of the loading. Because of the large impedance mismatch between the fiber bundle and the steel bars, however, full equilibrium cannot be established within the very initial linear-elastic portion of the pulse.

Throughout this paper, the elastic modulus *E* fitted from the initial, approximately linear portion of the stress–strain curves is an apparent engineering modulus rather than a true intrinsic material constant. Its absolute value carries systematic errors from several sources: (i) machine and fixture compliance, which underestimates the stiffness in quasi-static tests; (ii) deformation of the epoxy end-tabs and metal connectors, which adds a small series compliance in the dynamic specimens; and (iii) at medium and high strain rates, stress-wave superposition, dispersion, and radial/axial inertia of the filaments, which produce oscillations and apparent inertial stiffening. Consequently, the apparent modulus should not be interpreted as an intrinsic modulus increase in the fiber material; only the rate-dependent trend is physically meaningful and is used consistently in the subsequent model fitting.

### 2.3. Statistical Model and Constitutive Model

#### 2.3.1. Statistical Model Based on Weibull Distribution

To establish a statistical strength model for the fiber bundle, the following basic assumptions are required:

(i) Each individual filament behaves elastically before failure and follows Hooke’s law [19];
(ii) All filaments undergo the same axial strain, and interactions between fibers, such as friction and shear coupling, are neglected;
(iii) When some filaments break, the load originally carried by them is instantaneously and uniformly redistributed to the remaining intact filaments;
(iv) The strength of each filament is governed by randomly distributed microscopic defects, and its fracture strength follows a two-parameter Weibull distribution [20].

For a bundle of *N* filaments with cross-section *A*, under axial strain *ε*, the surviving filaments carry

*P* = *EεA*(*N* − *N_b_*) (1)

where *N_b_* is the number of broken filaments. Dividing by the total initial cross-section *N·A* gives the average bundle stress, in which the effect of the broken fibers enters through the survival fraction:

*σ*(*ε*) = *Eε*(1 − *N_b_*/*N*) = *Eε*(1 − *P_f_*(*ε*)) (2)

with the cumulative failure probability *P_f_*(*ε*) = *N_b_*/*N*. For a two-parameter Weibull strength distribution,

(3) $$P_{f}(\sigma )=1-e^{-{\left(\sigma /\sigma_{0}\right)}^{\beta }}$$

where *β* is the shape parameter and *σ***_0_** is the scale parameter. Substituting *σ* = *E*·*ε* into Equation (3) and the result into Equation (2) gives the bundle damage law:

(4) $$\sigma (\varepsilon )=\mathrm{E\varepsilon}\cdot e^{-{\left(\mathrm{E\varepsilon}/\sigma_{0}\right)}^{\beta }}$$

For parameter identification, Equation (3) is linearized. Because logarithms of dimensional quantities are not defined, all stresses are normalized by a reference stress *σ_ref_* (for which the quasi-static mean strength of the corresponding bundle is used). With the dimensionless variables *X* = ln(*σ*/*σ_ref_*) and *Y* = ln[−ln(1 − *P_f_*)], Equation (3) becomes

*Y* = *βX* − *β*ln(*σ*_0_/*σ_ref_*) (5)

so that *β* and *σ***_0_** follow from the slope and intercept. To verify whether the tensile strength follows a two-parameter Weibull distribution, the linearity of the Weibull transformation is examined using the coefficient of determination *R*^2^. If *R*^2^ is close to 1, the tensile strength is considered to conform to the two-parameter Weibull distribution.

#### 2.3.2. Macroscopic Empirical Constitutive Model Based on Ramberg–Osgood

The Ramberg–Osgood equation is a classic empirical model for describing the nonlinear stress–strain behavior of materials [17]. Owing to its simple form and good fitting capability, it has been widely used in engineering. The traditional equation expresses strain as a function of stress:

*ε* = (*σ*/*E*) + (*σ*/*K*)*^n^* (6)

The implicit form of Equation (6) requires iterative solution for stress, which is inconvenient for engineering analysis, and a closed-form inversion of the classical Ramberg–Osgood equation is available only in a piecewise form. To obtain an explicit relation that retains the same physical ingredients, consider the following explicit saturation-form R–O-type model (linear elasticity at small strain and a bounded stress level at large strain), which is consistent in spirit with the Ramberg–Osgood description:

(7) $$\varepsilon =\frac{\sigma }{E}{\left[1-{\left(\frac{\sigma }{K}\right)}^{n}\right]}^{-\frac{1}{n}}$$

Equation (7) exhibits two exact limiting behaviors. When the applied stress is well below *K*, the response asymptotically reduces to linear elasticity, with only a small power-law correction of the Ramberg–Osgood type. When the stress approaches *K* from below, the strain tends to infinity, so that *K* acts as a saturation stress. Equation (7) admits an exact closed-form inversion, from Equation (7),

(8) $${\left(\frac{\mathrm{E\varepsilon}}{\sigma }\right)}^{n}={\left[1-{\left(\frac{\sigma }{K}\right)}^{n}\right]}^{-1}$$

(9) $${\left(\frac{\sigma }{\mathrm{E\varepsilon}}\right)}^{n}=1-{\left(\frac{\sigma }{K}\right)}^{n}$$

(10) $${\left(\frac{\sigma }{K}\right)}^{n}=1-{\left(\frac{\sigma }{\mathrm{E\varepsilon}}\right)}^{n}$$

Substituting this back and solving for σ is given as Equation (11).

(11) $$\sigma =\mathrm{E\varepsilon}{\left[1+{\left(\frac{\mathrm{E\varepsilon}}{K}\right)}^{n}\right]}^{-\frac{1}{n}}$$

This inverse retains the physical ingredients of the classical Ramberg–Osgood law while avoiding its implicit form and piecewise inversion. Where *E* is the initial elastic modulus, *K* is a reference stress parameter related to the characteristic strength, and *n* characterizes the degree of nonlinearity. A larger *n* indicates weaker nonlinearity and a response closer to ideal linear elasticity, whereas a smaller *n* corresponds to more pronounced nonlinear softening. Compared with a first-order Taylor-type approximation, the present saturation form provides higher fitting accuracy for the nonlinear segment.

The Weibull statistical damage model and the Ramberg–Osgood model are complementary: the former describes progressive failure from microscopic statistical damage evolution, while the latter empirically captures the macroscopic nonlinear stress–strain response. In this study, the explicit saturation-form R–O-type model is used to fit the tensile stress–strain data of both carbon and aramid fiber bundles at different strain rates. For carbon fiber bundles, a unified parameter set is examined to describe their rate-insensitive behavior. For aramid fiber bundles, parameters are identified at each strain rate, and the rate dependence of the reference stress parameter *K* is explicitly considered.

## 3. Results and Discussion

### 3.1. Tensile Response and Damage Evolution at Different Strain Rates

Within the strain rate range of 10^−4^–10^2^ s^−1^, carbon fiber bundles and aramid fiber bundles exhibit fundamentally different tensile responses and damage evolution. Each test condition was repeated five times, and representative curves were selected for detailed analysis.

#### 3.1.1. Carbon Fiber Bundles

**Figure 4:**
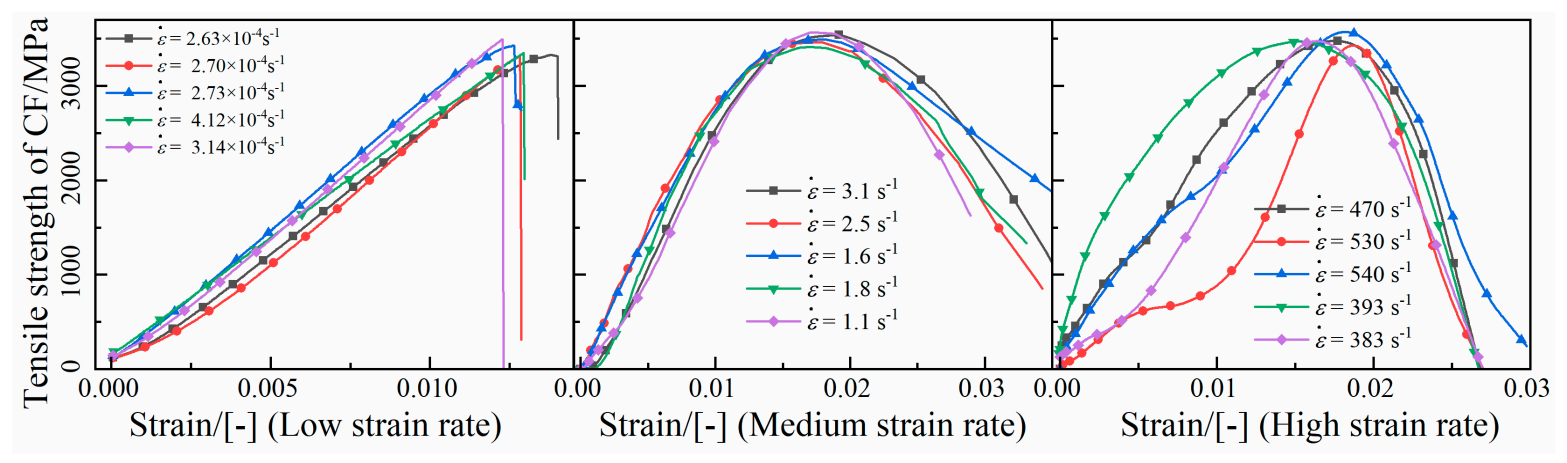
Tensile stress–strain curves of carbon fiber bundles at different strain rates.

**Figure 5:**
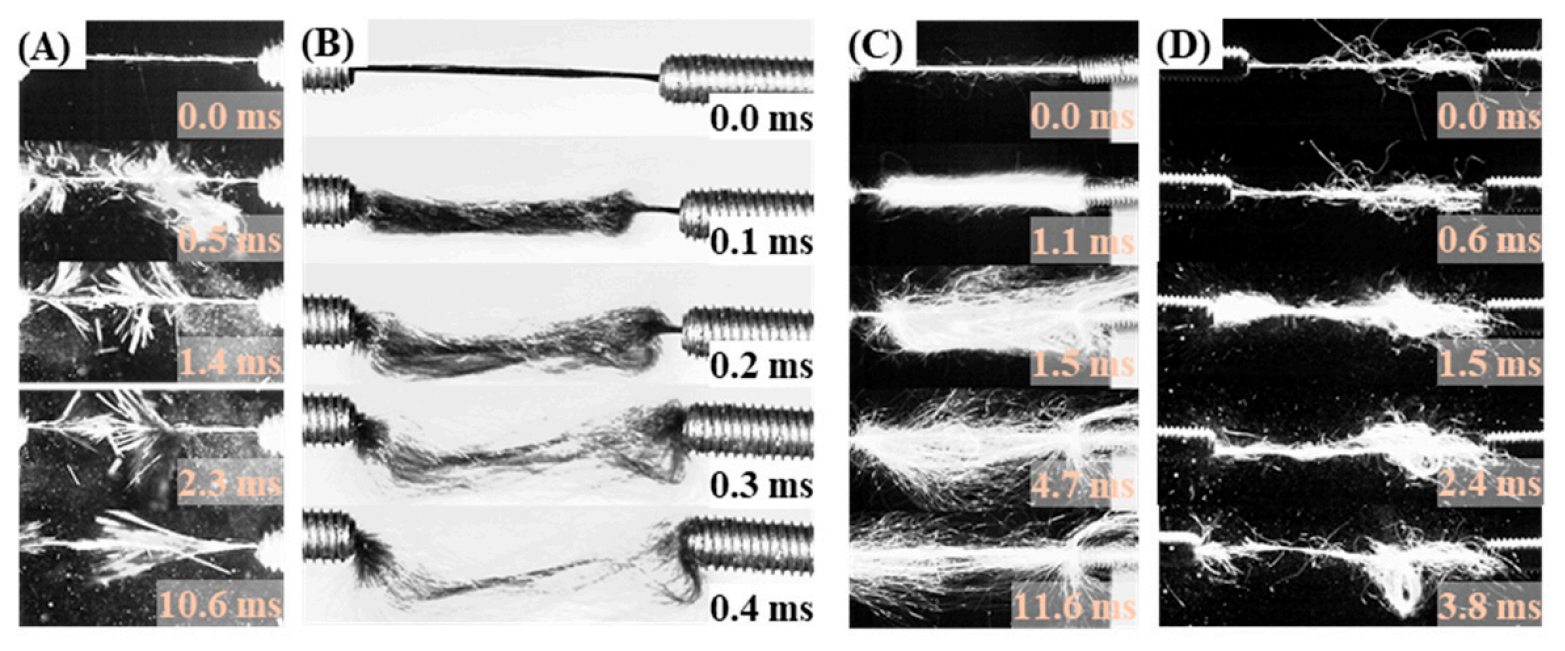
Failure processes of carbon fibers and aramid fibers under medium and high strain rates recorded by high-speed camera: (**A**) CF at 2.5 s^−1^; (**B**) CF at 530 s^−1^; (**C**) AF at 6.13 s^−1^; (**D**) AF at 492 s ^−1^. The strain rates shown here are the actual values of the representative tests, which are on the same order of magnitude as the average values listed in Table 2.

Carbon fiber bundles show pronounced strain-rate insensitivity. Under quasi-static loading (Figure 4), the stress–strain curves enter a linear elastic regime immediately upon loading and fracture instantaneously after the peak stress, with a slight curvature just before the peak in a few curves. The average tensile strength is 3375 MPa and the failure strain is 0.013, indicating typical brittle fracture. At intermediate strain rates (Figure 4), the curves remain approximately linear, and the average strength increases to 3496 MPa, about 3.6% higher than the quasi-static value. The gradual post-peak decline observed in some curves is attributed to signal interference during dynamic testing. High-speed camera images (Figure 5A) show that the fibers still fail progressively in a fiber-by-fiber manner and spring outward, but with greater localization than in the quasi-static case. At high strain rates (Figure 4), the ascending branch of the stress–strain curves remains linear, and the average strength is 3485 MPa, differing by only 0.3% from the intermediate rate, a negligible difference. High-speed images (Figure 5B) capture explosive fragmentation of the specimen within 0.4 ms, where a multitude of fibers reach their ultimate strain almost simultaneously. The failure mode transitions from progressive, sequential fiber failure to a catastrophic, synchronous fracture. Across the three strain rates, the tensile strength of carbon fiber bundles shows no statistically significant strain rate effect; only the degree of fracture synchronization and the spatiotemporal concentration of energy release increase with strain rate.

**Table 2:** Statistical results of tensile strength for carbon and aramid fiber bundles under different strain-rate conditions (*n* = 5 valid tests per condition).

Material	Strain Rate Range (s^−1^)	Average Strength (MPa)	Standard Deviation (MPa)
CF	2.63 × 10^−4^–4.12 × 10^−4^	3375	74
	1.1–3.1	3496	54
	383–540	3485	53
AF	2.34 × 10^−4^–4.08 × 10^−4^	1950	100
	0.313–0.825	2161	91
	197–536	3129	116

#### 3.1.2. Aramid Fiber Bundles

**Figure 6:**
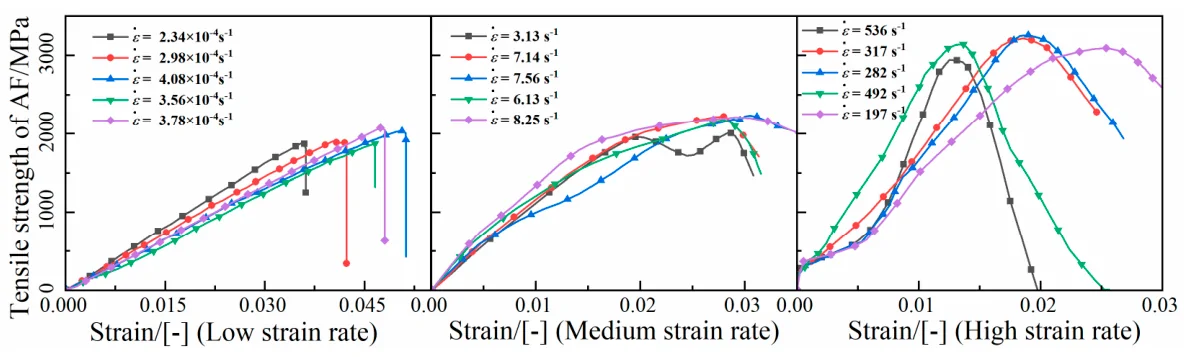
Tensile stress–strain curves of aramid fiber bundles at different strain rates.

In contrast, aramid fiber bundles exhibit significant strain rate strengthening. Under quasi-static loading (Figure 6), the stress–strain curves are approximately linear elastic over the entire loading history, with only a slight nonlinear deviation near the peak. The average tensile strength is 1950 MPa and the failure strain is 0.046; the fracture surfaces show a highly fibrillated morphology (Figure 5C,D). At intermediate strain rates (Figure 6), the tensile strength increases to 2161 MPa, an increase of 10.8% over the quasi-static value, while the failure strain decreases to ~0.035 (a reduction of 23.9%). The curves exhibit markedly enhanced nonlinearity and a softening trend. High-speed images (Figure 5D) display more concentrated and violent failure with intensified fiber splitting and dispersal. At high strain rates (Figure 6), the curves resume a nearly linear character. The strength further rises to 3129 MPa, representing a 60.5% increase from the quasi-static condition, and the failure strain drops to 0.020 (a decrease of 56.5%). High-speed images (Figure 6) indicate that the failure mode transforms into a transient “elongation–fibrillation–overall rupture” process, with near-simultaneous fiber fracture and a more pronounced brittle appearance on the fracture surface. The strength of aramid fibers increases monotonically with strain rate (overall increase 60.5%), while the failure strain decreases monotonically; the failure mode evolves from quasi-ductile splitting to brittle fracture.

The irregular features observed in some high-rate curves (Figure 4 and Figure 5)—nonlinear ascending branch, rounded peak, and local post-peak oscillation—are not expressions of intrinsic material softening. They result from combined wave dispersion, inertial effects, and insufficient time for progressive damage redistribution. In SHTB tests, the superposition and dispersion of the incident, reflected, and transmitted waves distort the early response, especially for loosely coupled filaments whose load transfer is itself wave-mediated. At strain rates of the order of 10^2^ s^−1^, radial and axial inertia of the filaments cause apparent inertial stiffening (see the definition of the apparent modulus in Section 2.2) and delay the peak. Moreover, load redistribution between filaments by friction and shear coupling cannot keep pace with the imposed loading rate, so a large fraction of the filaments reaches its ultimate strain almost simultaneously and the nonlinear region broadens; for the aramid bundle, the partial release and re-engagement of split microfibrils within a few microseconds further roughens the post-peak traces. Despite these effects, the mean strengths remain reliable because the scatter bands of the five repeated tests overlap strongly.

In summary, within the 10^−4^–10^2^ s^−1^ range, carbon fiber bundles exhibit no strain-rate effect on tensile strength, and the damage mode only involves a change in the degree of synchronization of brittle fracture. Aramid fiber bundles, by contrast, display significant positive strain-rate sensitivity, with a strength increase exceeding 60%. Their mechanical response and damage evolution must be explicitly accounted for in dynamic loading analyses.

### 3.2. Analysis of the Strain Rate Effect

**Figure 7:**
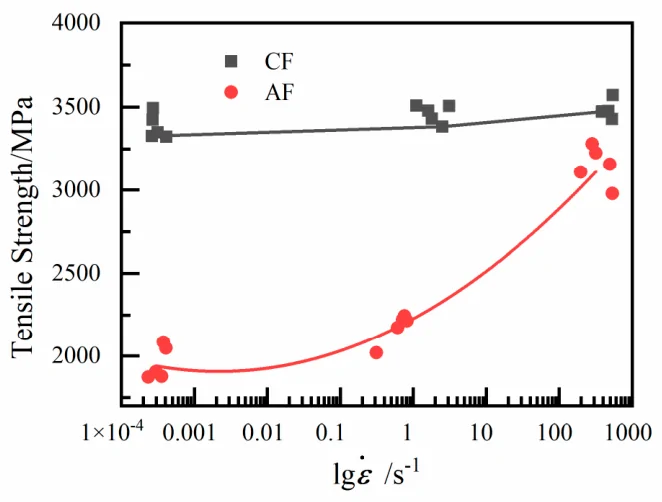
Comparison of tensile strength of carbon and aramid fiber bundles as a function of logarithmic strain rate.

Figure 7 compares the tensile strength of carbon and aramid fiber bundles as a function of logarithmic strain rate, and Table 2 summarizes the corresponding statistical analysis (strain-rate ranges, mean strength, and standard deviation; five valid tests per condition, i.e., 30 valid tests in total). The two fiber types exhibit distinctly different rate sensitivity over the range of 10^−4^–10^2^ s^−1^. The strength of carbon fiber bundles remains essentially unchanged with strain rate. The quasi-static strength is 3375 MPa, which becomes 3496 MPa at the intermediate strain rate and 3485 MPa at the high strain rate. The maximum relative deviation among the three data points is only 3.6%, falling within the experimental scatter (Table 2), which indicates no significant rate dependence.

In contrast, aramid fiber bundles display pronounced nonlinear positive strain rate sensitivity. The strength increases monotonically with $\mathrm{lg}\dot{\varepsilon }$, and the increment is sharply amplified in the high strain rate regime: from 1950 MPa under quasi-static loading to 2161 MPa at the intermediate rate, an increase of 10.8%, and further to 3129 MPa at the high rate, an increase of 60.5%. Using $\mathrm{lg}\dot{\varepsilon }$ as the independent variable, the strength variation within the investigated range can be empirically described by a quadratic polynomial:

(12) $$\sigma =45.10{\left(\mathrm{lg}\dot{\varepsilon }\right)}^{2}+240.15(\mathrm{lg}\dot{\varepsilon })+2226.89$$

Equation (12) was fitted to all 15 individual data points within the investigated strain-rate range. It explains ~95.7% of the strength variation (*R*^2^ = 0.957), with an RMSE of ~122 MPa. The positive quadratic coefficient (45.10 MPa) suggests nonlinear strengthening: the strength gain per unit logarithmic strain-rate increment increases at higher strain rates. The fit is valid only for interpolation within the tested range; extrapolation should be avoided. In summary, carbon fiber bundles are rate-independent, whereas aramid fiber bundles exhibit nonlinear strength enhancement that accelerates with strain rate, with a total increase exceeding 60%. A rate-dependent constitutive description is therefore required for aramid fiber bundles in composite analysis.

### 3.3. Statistical Damage Analysis Based on Weibull Distribution

Based on the tensile test data at each strain rate, the stable rising portion of the stress–strain curves was selected for two-parameter Weibull fitting, and the statistical damage constitutive equation was used to generate theoretical curves to verify the applicability of the model.

**Figure 8:**
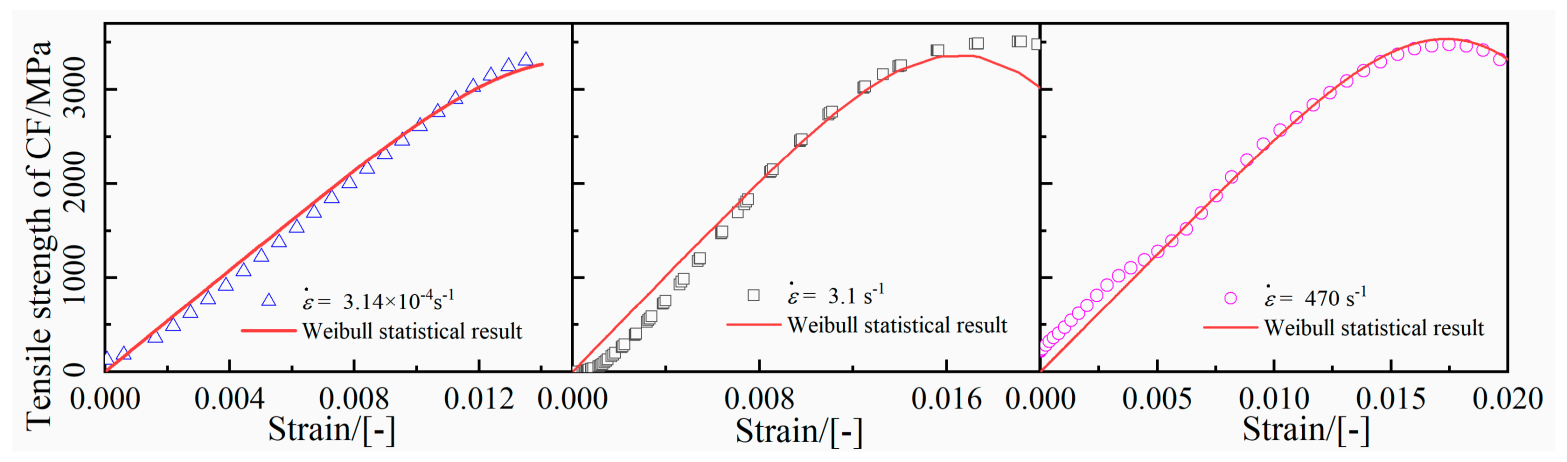
Weibull statistical results of carbon fiber bundles at different strain rates.

**Figure 9:**
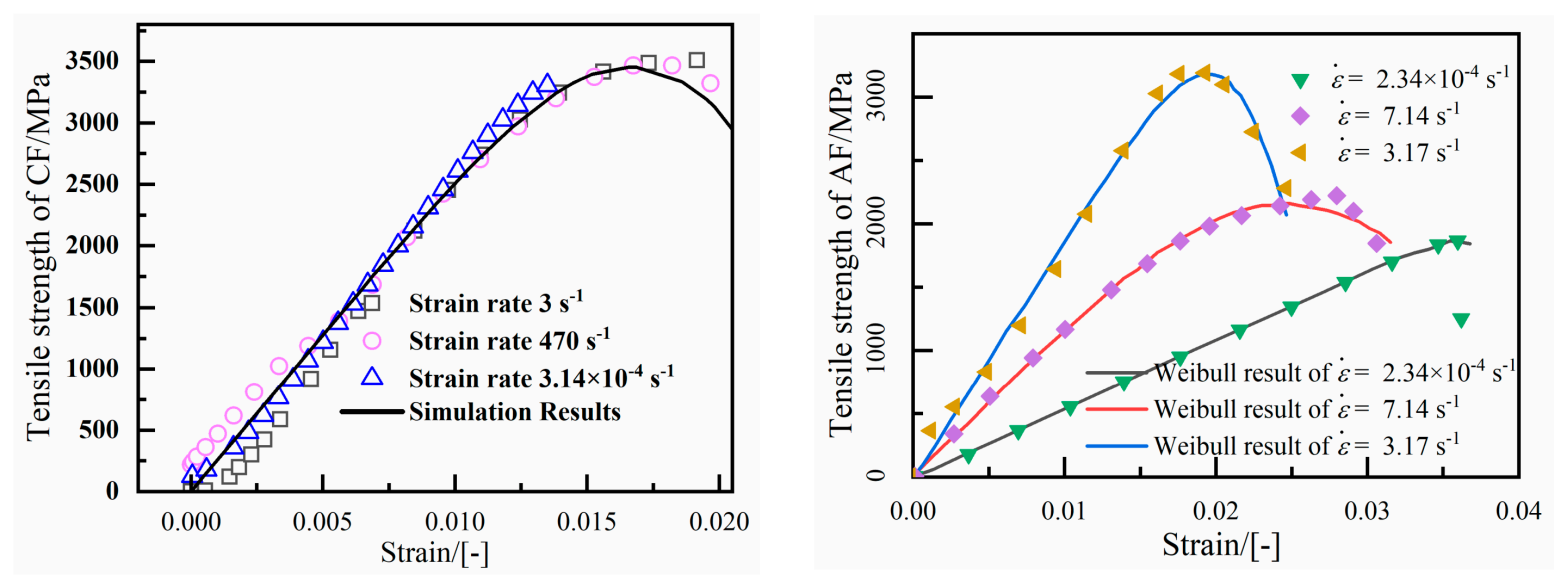
Comparison of Weibull statistical results of carbon fiber and aramid fiber bundles at different strain rates.

For carbon fiber bundles, the shape parameter obtained under quasi-static loading is *β* = 4.7, the scale parameter *σ**_0_*** = 5400 MPa, with a coefficient of determination *R^2^* = 0.89. The theoretical curve agrees well with the experimental curve in the ascending branch before the peak, as shown in Figure 8. At the intermediate strain rate, *β* = 5.0, *σ***_0_** = 5700 MPa, and *R*^2^ = 0.95, close to the quasi-static values; at the high strain rate, *β* = 4.8, *σ***_0_** = 6000 MPa, and *R*^2^ = 0.93. Over the three strain rates, *β* remains within 4.7–5.0 and *σ***_0_** within 5400–6000 MPa, exhibiting no systematic variation, which indicates that the strength distribution characteristics do not change with strain rate. A unified linear regression using all data yields *β* = 4.9 and *σ***_0_** = 5900 MPa, consistent with the individually fitted parameters at each strain rate, further confirming the rate independence of the micro-defect distribution. Substituting the unified parameters into the constitutive equation gives theoretical curves that match the ascending portions of the experimental curves at different strain rates well, as shown in Figure 9, demonstrating the capability of the two-parameter Weibull model to describe the tensile behavior of carbon fiber bundles uniformly over the range of 10^−4^–10^2^ s^−1^. These model parameters can be embedded into microscopic representative volume elements, simplifying the constitutive description of the reinforcement and providing key parameters for cross-scale dynamic predictions of composites.

For aramid fiber bundles, the Weibull parameters change significantly with strain rate. Under quasi-static loading, *β* = 13.0, *σ***_0_** = 2500 MPa, *R*^2^ = 0.99, and the strength distribution is concentrated. At the intermediate strain rate, *β* decreases to 3.5, *σ***_0_** rises to 4100 MPa, *R*^2^ = 0.99, and the scatter increases. At the high strain rate, *β* rebounds to 7.6 and *σ***_0_** further increases to 4700 MPa, *R*^2^ = 0.98. The scale parameter *σ***_0_** increases monotonically, while *β* exhibits a non-monotonic trend of first decreasing and then increasing, reflecting the complex change in failure synchronization with strain rate. For the shape parameter β, it can be treated as a constant within a specific strain rate interval in practical applications. Substituting the rate-dependent scale parameter *σ***_0_** into the modified model yields theoretical curves that agree well with the experimental data under quasi-static, intermediate, and high strain rates, as shown in Figure 9, verifying the validity of the modified model.

In summary, the Weibull parameters of carbon fiber bundles remain constant over the entire strain-rate range, indicating a statistically rate-independent material. In contrast, both the scale and shape parameters of aramid fiber bundles vary significantly with strain rate, and a rate-dependent statistical damage model is required to describe their mechanical behavior.

### 3.4. Based on the Macroscopic Constitutive Model of Ramberg–Osgood

**Figure 10:**
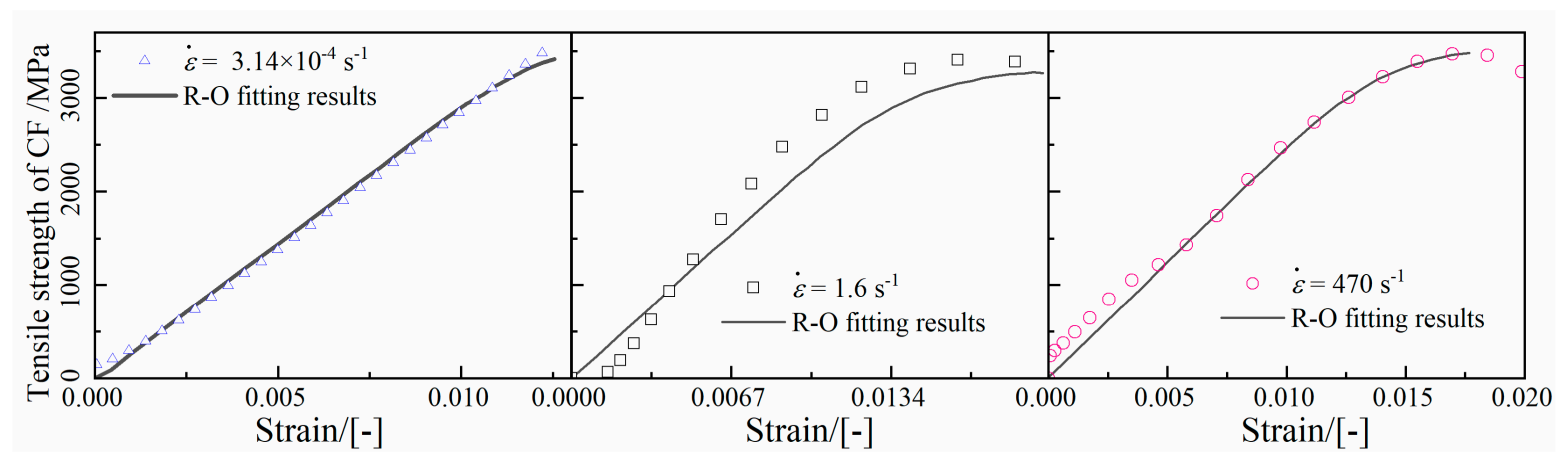
Ramberg–Osgood fitting results for carbon fiber bundles at different strain rates.

The Ramberg–Osgood macroscopic empirical model describes the nonlinear stress–strain response from the perspective of continuum mechanics with a small number of parameters, offering intuitive physical interpretation and ease of engineering application. The second-order explicit approximation of the Ramberg–Osgood model introduced in Section 2.3 was used to fit the experimental stress–strain curves of carbon and aramid fiber bundles at various strain rates. The fitting results and parameters for carbon fiber bundles are presented in Figure 10 and Table 3. The coefficients of determination *R*^2^ are 0.99, 0.99, and 0.98 for quasi-static, intermediate, and high strain rates, indicating that the three-parameter model can characterize the tensile nonlinear behavior of carbon fiber bundles with high accuracy over a wide range of strain rates. Among the three parameters, attention is focused on the strength-related parameter *K* and the shape parameter n. The values of *K* are 3.6 GPa, 3.3 GPa, and 3.6 GPa at quasi-static, intermediate, and high strain rates, respectively; these variations fall within the experimental scatter, corroborating the rate-independent strength conclusion drawn from the Weibull analysis. The shape parameter *n* exhibits pronounced rate sensitivity: it equals 11.42 under quasi-static loading and decreases to 7.19 and 7.66 at intermediate and high strain rates. A larger *n* value corresponds to weaker nonlinear softening, i.e., a response closer to linear elasticity. Under quasi-static loading, fibers fail progressively in a fiber-by-fiber manner with continuous load redistribution, and the curve deviates slightly from linearity only near the peak. Under dynamic loading, fiber failure tends to synchronize, with a large number of filaments reaching their ultimate strain almost simultaneously, causing the nonlinear region to expand significantly and *n* to decrease accordingly. Thus, the variation in *n* essentially provides a mathematical mapping of the degree of failure synchronization onto the macroscopic mechanical response and can serve as an indirect indicator for distinguishing progressive failure from synchronous failure.

**Figure 11:**
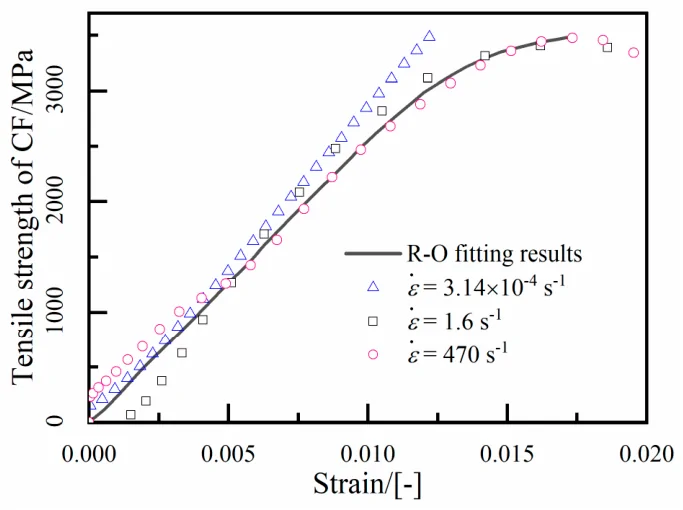
Unified Ramberg–Osgood fitting results for carbon fiber bundles at different strain rates.

Meaning and role of the unified fit. Because the strength of the carbon fiber bundle is rate-insensitive while the curve shape is not (*n* varies with failure synchronization), a single (unified) *R–O* parameter set was additionally identified by simultaneously fitting all fifteen CF curves over 10^−4^–10^2^ s^−1^. The unified fit yields *E* = 257.7 GPa, *K* = 3.5 GPa, and *n* = 8.76 (Table 3). Its mathematical and physical meaning is a rate-averaged compromise: *E* is fixed at the apparent quasi-static value, *K* takes an intermediate value representative of the rate-independent strength, and *n* = 8.76 is an average of the rate-wise values rather than a new material constant. The result is shown in Figure 11.

The R–O model fitting results for aramid fiber bundles are also listed in Table 3. Owing to their pronounced strain-rate sensitivity, all parameters vary with strain rate to different extents. The apparent modulus of the AF bundle rises from 45.7 GPa (quasi-static) to 132.4 GPa (intermediate) and 200.7 GPa (high rate). For comparison, tensile moduli of about 70–115 GPa are typically reported for Kevlar-49-type filaments at quasi-static rates, and appreciable rate-dependent stiffening of aramid yarns and bundles has been documented by several authors [9,21,22]. The absolute values obtained here exceed the filament-level values, and we attribute this gap to the bundle-level effects quantified in Section 2.2: machine/fixture and end-tab compliance (dominant at low rate, causing an underestimate), and stress-wave oscillation, wave dispersion, and radial/axial filament inertia (dominant at high rate, causing a systematic overestimate when the modulus is fitted from the oscillatory rising branch). The grip load-transfer through metal threaded connectors can further amplify the apparent stiffness. Because the same protocol was used for all rates, these artifacts shift the absolute values but cannot create the observed monotone trend— the same artifacts acting on the identically prepared and tested carbon fiber bundle produce no significant modulus change. We therefore use the apparent *E* only as a consistent modeling input within this study and recommend a dedicated compliance-corrected, gauge-length-controlled campaign before the high-rate modulus is used as an intrinsic design input.

**Figure 12:**
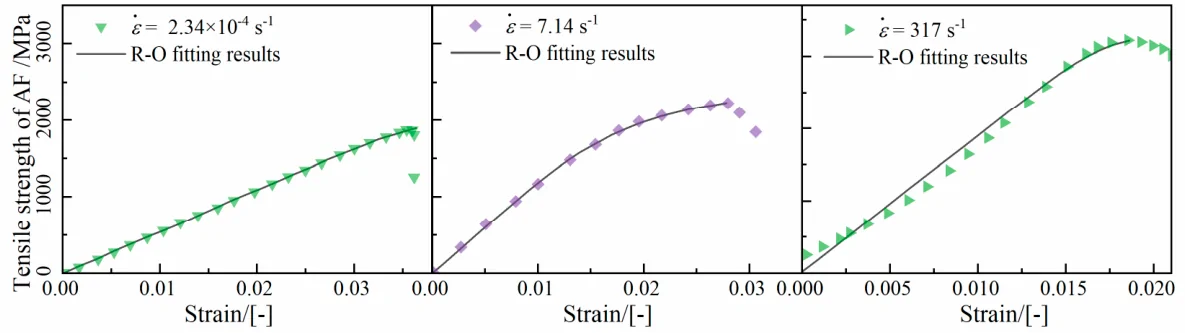
Ramberg–Osgood fitting results for aramid fiber bundles at different strain rates.

The reference stress parameter *K* of the aramid bundle, which is directly related to the tensile strength, exhibits the most critical rate dependence. Based on the fitted data, the rate-dependent expression for *K* is obtained as

(13) $$K(\dot{\varepsilon })=1.932+0.043\cdot e^{\frac{\ln(\dot{\varepsilon }/{\dot{\varepsilon }}_{0})}{4.148}}=1.932+0.043\cdot {\left(\frac{\dot{\varepsilon }}{{\dot{\varepsilon }}_{0}}\right)}^{\frac{1}{4.148}}$$

where *K* is in GPa, ${\dot{\varepsilon }}_{0}$ = 2.34 × 10^−4^ s^−1^ is the reference (quasi-static) strain rate, and the argument of the exponential is the dimensionless logarithm of the dimensionless strain-rate ratio, so Equation (13) is dimensionally consistent. Equivalently, *K*-1.932 grows exponentially with lg($\dot{\varepsilon }/{\dot{\varepsilon }}_{0}$), i.e., as a power law of the dimensionless strain rate $\dot{\varepsilon }/{\dot{\varepsilon }}_{0}$. The coefficient of determination for this fitting is *R*^2^ = 0.99. By combining the experimentally determined apparent *E* and *n* at a given strain rate with the corresponding *K* from Equation (13), the complete stress–strain response under that loading condition can be obtained. Figure 12 compares the predicted curves with the experimental data; good agreement is observed throughout the entire loading process, confirming the accuracy of the model.

### 3.5. Comparison of the Three Rate Descriptions (R–O, Johnson–Cook, Cowper–Symonds)

To assess whether the viscoplastic rate laws of Johnson–Cook (JC) and Cowper–Symonds (CS) could replace the Ramberg–Osgood (R–O) description adopted in this study, the three formulations are compared at the level of their common capability, namely the prediction of the strain-rate-dependent tensile strength. Two reasons motivate a strength-level comparison. First, the JC and CS rate terms are strength relations and do not define a stress–strain response; constructing full curves for them would require an arbitrary choice of a baseline shape function, which would bias the comparison. Second, and more fundamentally, JC and CS were developed for elastic–plastic metals in which strain-rate sensitivity enters as a multiplicative strengthening of a plastic flow stress. The present fiber bundles show no plasticity: they respond in a nonlinear-elastic manner up to a damage-controlled peak and then fail. A multiplicative strength scalar is therefore a poorer conceptual match than an explicit nonlinear-elastic form such as the R–O model (Equation (11)), which reproduces the complete curve with three parameters; the quantitative comparison below confirms this at the strength level.

The Johnson–Cook strain-rate term adopted for comparison is (in isothermal form) [23]:

(14) $$\sigma_{\mathrm{JC}}(\dot{\varepsilon })=\sigma_{0}\cdot [1+C\cdot \ln(\dot{\varepsilon }/{\dot{\varepsilon }}_{0})]$$

where *σ***_0_** = 1950 MPa is the quasi-static mean strength and ${\dot{\varepsilon }}_{0}$ = 2.34 × 10^−4^ s^−1^ the reference strain rate. The single material constant *C* = 0.0317 was identified by least-squares minimization of the residuals between Equation (14) and the three measured mean strengths (1950, 2161 and 3129 MPa).

The Cowper–Symonds law adopted for comparison is as follows [24]:

(15) $$\sigma_{\mathrm{CS}}(\dot{\varepsilon })=\sigma_{0}\cdot [1+{\left(\dot{\varepsilon }/D\right)}^{1/P}]$$

with the two material constants *D* = 998 s^−1^ and *p* = 2.272 identified by the same least-squares procedure. The Ramberg–Osgood prediction of strength is obtained by substituting the rate-dependent *K*($\dot{\varepsilon }$) from Equation (13), together with the rate-wise apparent *E* and *n*, into Equation (11) for each of the five repeated tests at a given strain rate, and taking the maximum of each fitted curve; the reported R–O strength is therefore the mean ± SD of the five curve-level predictions at each strain rate. The comparison is collected in Table 4.

The error columns of the three models have different meanings and should be read differently. The *JC* and *CS* columns contain no scatter because these laws are fitted directly and only to the three mean strengths: once the global constant *C* (or the pair *D*, *p*) is identified, each strain rate yields exactly one predicted strength, with no curve-level identification step. Their deviations from the measured means are therefore purely systematic (model-form) errors: *JC* is non-zero because a single logarithmic coefficient cannot follow the upward-convex strengthening of aramid (hence +19.2% at 7.14 s^−1^ and −9.7% at 317 s^−1^), while *CS* is (near-)zero only because two parameters fitted to three points reproduce them by construction, with zero remaining degrees of freedom. The *R–O* column, in contrast, is accompanied by a standard deviation because the model is fitted to each individual curve: the five predictions at one strain rate inherit the test-to-test scatter of the raw data through the curve-fitting step. The sign of the mean error is systematically negative (−3.8%, −0.9%, −4.2%) because the *R–O* ascending branch at a prescribed modulus rounds off before the measured peak—the onset of damage softening near the peak lies outside the capacity of the three-parameter form—so the fitted curve attains its maximum slightly below the measured strength. In short, *JC/CS* errors are deterministic model-form errors of a global strength law, whereas the *R–O* error is a curve-level statistical quantity (mean ± SD) whose small negative bias reflects the known near-peak limitation discussed in Section 3.4. For engineering use, the *R–O* description is preferable for these fibers because it provides complete stress–strain curves (not only strength scalars) with the lowest curve-level error and with all parameters identified from raw data rather than from three mean points.

## 4. Conclusions

This study compared the tensile behavior of carbon fiber (CF) and aramid fiber (AF) bundles over 10^−4^ to 10^2^ s^−1^, using quasi-static, pneumatic impact, and split Hopkinson tensile bar tests. The main conclusions are as follows:

(1) CF bundles are rate-insensitive in strength, with failure changing from progressive to nearly simultaneous fracture. AF bundles show pronounced strengthening: strength increases from 1950 to 3129 MPa (+60.5%), the apparent modulus increases from 45.7 to 200.7 GPa (an apparent, protocol-dependent quantity affected by compliance and wave/inertia effects), and the failure strain decreases from 0.046 to 0.020, with failure shifting from quasi-ductile splitting to brittle fracture.
(2) The Weibull model describes the damage evolution well. CF parameters *β* and *σ_0_* remain nearly constant. For AF, *σ_0_* increases linearly with logarithmic strain rate, while *β* is non-monotonic. The modified model with rate-dependent *σ*_0_ predicts AF responses accurately.
(3) The Ramberg–Osgood model fits both fiber types. For CF, a unified, rate-averaged parameter set (*E* = 257.7 GPa, *K* = 3.5 GPa, *n* = 8.76) is applicable to strength-level engineering predictions, with *n* decreasing from 11.42 to 7.19–7.66 under dynamic loading; rate-wise sets should be used when the nonlinear curve shape matters. For AF bundles, the apparent *E* increases with strain rate, and *K* grows as a power law of the dimensionless strain rate $\dot{\varepsilon }/{\dot{\varepsilon }}_{0}$ (equivalently, exponentially with lg($\dot{\varepsilon }/{\dot{\varepsilon }}_{0}$)), enabling rate-dependent prediction of the complete tensile response. At the strength-prediction level, this description is more accurate for aramid than the Johnson–Cook law and is physically more informative than the Cowper–Symonds law, which reproduces the three mean strengths by construction.
(4) For carbon fiber bundles, the fiber strength rate effect can be neglected in this range, but matrix/interface effects and failure-mode changes should be considered when extending to composites. For aramid fiber bundles, rate-dependent constitutive models are required. The models adopted and parameterized in this study provide reliable material input for impact-resistant design of fiber bundles and a reference for their composites within the tested range.

## Figures and Tables

**Table 1:** Basic material data of the tested fiber bundles.

Property	TZ300 Carbon Fiber Bundle (CF)	P240 Aramid Fiber Bundle (AF)
Supplier	Weihai Guangwei Composite Materials Co., Ltd. (Weihai, China)	Zhongfang New Materials Technology Co., Ltd. (Dongying, China)
Specification	3 K	3 K
Nominal bundle diameter (mm)	0.28	0.20
Gauge length (mm)	Quasi-static: 40 ± 2.0; intermediate/high strain rate: 20 ± 1.0	

**Table 3:** Fitted Ramberg–Osgood parameters for carbon fiber and aramid fiber bundles.

Project	Strain Rate (s^−1^)	*E* (GPa)	*K* (GPa)	*n*	*R* ^2^
Carbon Fiber	3.14 × 10^−4^	257.7	3.6	11.42	0.99
Carbon Fiber	1.03	257.7	3.3	7.19	0.99
Carbon Fiber	470	257.7	3.6	7.66	0.98
Carbon Fiber (unified fit)	10^−4^–10^2^	257.7	3.5	8.76	/
Aramid Fiber	2.34 × 10^−4^	45.7	1.98	15.29	0.99
	7.14	132.4	2.39	3.68	0.99
	317	200.7	3.22	12.82	0.98

**Table 4:** Strength-level comparison of the three rate descriptions (aramid).

Strain Rate (s^−1^)	Test Peak (MPa)	JC (MPa)	CS (MPa)	R–O
Low strain rate	1950 ± 100	1950 (0.0%)	1950 (0.0%)	1877 ± 191
Medium strain rate	2161 ± 91	2590 (+19.2%)	2161 (0.0%)	2154 ± 151
High strain rate	3129 ± 116	2824 (−9.7%)	3129 (0.0%)	2997 ± 351
RMSE (MPa)	\	298 (12.3%)	0	88 (3.6%)

## Data Availability

The original contributions presented in this study are included in the article. Further inquiries can be directed to the corresponding author.
